# Sensor Integration in a Low Cost Land Mobile Mapping System

**DOI:** 10.3390/s120302935

**Published:** 2012-03-02

**Authors:** Sergio Madeira, José A. Gonçalves, Luísa Bastos

**Affiliations:** 1 Universidade de Tras-Os-Montes e Alto Douro, Apartado 1013, 5000-801 Vila Real, Portugal; 2 CIMAR/CIIMAR, Centro Interdisciplinar de Investigação Marinha e Ambiental, Universidade do Porto, Rua dos Bragas, 289, 4050-123 Porto, Portugal; E-Mails: jagoncal@fc.up.pt (J.A.G.); lcbastos@fc.up.pt (L.B.); 3 Departamento de Geociências, Ambiente e Ordenamento do Território, Faculdade de Ciências da, Universidade do Porto, Rua Campo Alegre 687, 4169-007 Porto, Portugal

**Keywords:** mobile mapping system, digital camera, camera calibration, global navigation satellite systems, inertial navigation system

## Abstract

Mobile mapping is a multidisciplinary technique which requires several dedicated equipment, calibration procedures that must be as rigorous as possible, time synchronization of all acquired data and software for data processing and extraction of additional information. To decrease the cost and complexity of Mobile Mapping Systems (MMS), the use of less expensive sensors and the simplification of procedures for calibration and data acquisition are mandatory features. This article refers to the use of MMS technology, focusing on the main aspects that need to be addressed to guarantee proper data acquisition and describing the way those aspects were handled in a terrestrial MMS developed at the University of Porto. In this case the main aim was to implement a low cost system while maintaining good quality standards of the acquired georeferenced information. The results discussed here show that this goal has been achieved.

## 1. Introduction

A Mobile Mapping System (MMS) can be defined as a moving platform, over which a Direct Georeferencing System (DGS) and remote sensors are placed to acquire synchronized, related to time, data, in order to allow the determination of position and orientation of the platform and 3D positions of the objects captured by the remote sensors, and all necessary software tools used to process, analyze, classify, manipulate, store and update raw data and processed information. In particular, the final products of a MMS may include modeled results of road geometry (such as the centreline alignment in the form of design elements) and qualitative information of asset inventory (such as for traffic signs or pavement, the type of material, condition, *etc*.). A more detailed description of this technology can be found in [1–3].

**Figure 1.**
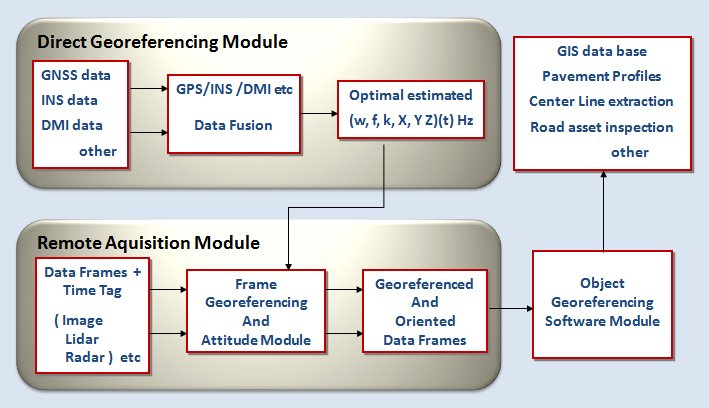
Flow chart of data acquisition and management in a MMS.

The remote sensors can be video cameras, laser scanners, *etc*. The DGS are, usually, associations of Global Navigation Satellite Systems (GNSS) receivers and Inertial Measurement Units (IMU), although other associations of dead reckoning devices that include odometers, inclinometers or digital compasses can also be used. The linkage of absolute positions and orientation parameters (obtained by the DGS) to the data obtained by the remote sensors allows for positional and geometrical information of the observed objects. Figure 1 shows a typical flowchart of the various modules used in a MMS.

It must be stressed that the accuracy requirements for orientation angles in a land based MMS, when compared to the airborne case is much smaller while providing the same positional accuracy of surveyed objects. This results from the fact that distances between the sensors and the observed objects are normally much smaller in a land MMS, and is an advantage because lower cost equipment can be used. However, airborne mobile mapping can acquire information over much larger areas in far less time, being therefore more effective for certain applications.

The achieved accuracy depends, to a large extent, on the quality of the DGS used. There is also some error propagation from the remote sensors which increases with object distance and depends mainly on imaging sensors quality and accuracy of the attitude angles. In a land MMS decimeter accuracy or better can be achieved. However, in urban environments, due to poor conditions of GNSS signal reception, the achieved accuracy necessarily decreases, sometimes to a few meters.

Over the last two decades several research groups have developed MMS [4,5] and nowadays commercial systems already exist, developed by renowned companies in the field (Riegl, Applanix, Topcon, *etc*.). Other commercial developments are referred in [6–8]. These systems are however, in general, quite expensive, and their installation and field operation is usually not straightforward. In recent years, with the progress of electronics and informatics, cheaper and easier to operate systems have been developed. The description of some existing low cost systems can be consulted through the corresponding references [9–15].

In this paper we focus on a MMS developed at the University of Porto. This system intends to be low-cost and easy to use and to install/uninstall in different types of moving platforms (terrestrial, aerial *etc*.). We start, in Section 2, with a brief introduction to Direct Georeferencing Systems, followed by a summary on the main aspects that need to be solved for a proper and robust implementation, on Section 3. In Section 4 we present the details of our specific implementation including dedicated calibration methods. Finally some results from the validation tests are presented and conclusions on the usefulness and efficiency of the U. Porto MMS are presented.

## 2. Direct Georeferencing System

A central part of a MMS is its Direct Georeferencing System (DGS) that provides platform position and attitude at a predefined time rate. DGS are composed by sensors that are able to give position and attitude data. GNSS and INS technology are central to a DGS.

A classic configuration for a DGS is the combination of GNSS receivers with an Inertial Measuring Unit. Due to the complementarity of GNSS and INS this combination can provide very high accuracy [7,8]. However other sensor combinations can be used (or added) in order to decrease the costs or increase the accuracy.

### 2.1. Inertial Navigation Systems (INS)

**Figure 2.**
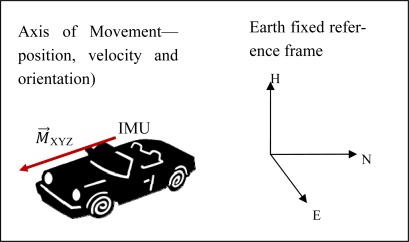
Inertial measuring principle.

Inertial navigation is a technique that allows the determination of position and orientation of a mobile body in a navigation (fixed) reference frame, by measuring its accelerations and angular velocities. Through successive mathematical integrations with respect to time, we can obtain velocity, position and attitude of the mobile platform. An Inertial Measuring Unit (IMU) contains accelerometers and gyroscopes, that together allow obtaining linear accelerations and angular velocities along three axes in a predefined inertial frame (for detailed information see, for example [16]). As a result, a motion vector for each instant can be obtained (illustrated in Figure 2 as vector *M⃗*).

An Inertial Navigation System (INS) has an IMU as main unit and additional hardware and software components that allow system setup and data processing leading to precise estimates of the position and attitude of the moving platform [16].

### 2.2. Global Navigation Satellite Systems (GNSS)

A description of GNSS is not within the scope of this work since there is enough and accessible literature on the subject (see for example [17]). In brief, GNSS is a system that allows one to obtain global coordinates, using electronic devices that analyze radio signals sent by a constellation of satellites. There are currently several of these systems in operation or under development [11,12]. The most widely used up to now is the American Global Positioning System (GPS). Currently the majority of the existing receivers use satellite positioning signals sent by this constellation. Receivers prepared to use also signals sent by GLONASS and GALILEO are also already in the market. Nowadays the trend in the quality and efficiency of GNSS systems is to improve, enhanced also by the interoperability between different systems, and sub-decimetric accuracies are already possible in kinematic observations, see for example [18].

### 2.3. GNSS/INS Integration

The advent of GPS during the eighties, allowed INS to be supplemented with the ideal type of information (coordinates without degradation over time) freeing it from the accumulation of errors when in a purely kinematic (*i.e.*, without updates during stops) mode. The use of a Kalman filter to integrate the two data sets is a common practice today, allowing the implementation of reliable and robust navigation systems [19–21].

The filter developed by Kalman [22,23] is essentially a least-squares solution. In a way the filter tries to combine all available data from observations (GNSS receivers, INS, odometers, *etc*.) and the knowledge about the vehicle dynamics and its measurement devices to provide an optimal estimate. The process starts from an initial estimate that is propagated in time using a system model, until an observation is made. Using the information from the observation, a new (optimal) estimate is defined. This new estimate is subsequently propagated in time until the next observation becomes available. So a clear predictor-corrector structure can be recognized and the filter can be seen to consist of two basic steps: a time update (or “prediction”) step that propagates the estimate in time and an observation update (or “correction”) step that inserts the observation information.

**Figure 3.**
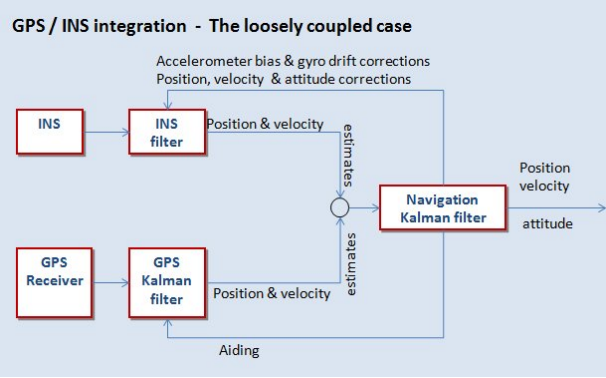
GPS/INS integration using Kalman filter. The loosely coupled case.

Various strategies or modes have been developed to integrate the data streams from the IMU and GNSS receiver, each based on a different way of how the data from the instruments are processed [9]. Figure 3 shows a diagram with the loosely coupled integration of GPS receiver and IMU measures, by means of a Kalman filter.

### 2.4. Other DGS Sensors

New developments of MMS should focus on using simpler and cheaper technology to facilitate and extend their use. New developments have occurred in this direction, not necessarily decreasing positional accuracy standards in the resulting spatial data. Sensors that are also used in Direct Georeferencing, besides GNSS receivers and INS, are, for example, one axis gyroscopes, odometers and digital compasses. An example is a GNSS plus dead reckoning commercial system, from U-blox company, that has as complementary components a one axis gyroscope device and connection to car odometer.

## 3. Aspects to Address in the Implementation of Mobile Mapping Systems

Once the survey platform is moving (*i.e.*, during a survey mission) time synchronization between acquired data, somewhere between a hundredth second and a millisecond, has to be assured. The usual approach consists in using a dedicated computer, or datalogger, to store and synchronize all the acquired data, in the same time frame. Other important aspect in mobile mapping is the overall calibration of the system that includes sensor calibration and the determination of relative orientation parameters between components. These are key issues in the MMS development at the University of Porto, and we will address these aspects, in more detail, in the following sub-sections.

### 3.1. Data Logging

Data compression, transfer and storage is usually a highly demanding task due to the large data volumes that can be produced by the sensors (video images, or laser), or by a high rate INS. Besides problems related with hardware delays (which affect the accurate time tag of the acquired information), there is a major concern associated with the logging capabilities of the computers, including data transfer velocity and storage capacity.

In a MMS survey the relatively large size of acquired data frames (like images or scan profiles) and the used frequency rates (normally several per second) can easily lead a computer system to its limits. However, due to the great evolution of informatics technology during the last two decades, noteworthy in storage, bus protocols and CPU (Central Processing Unit), nowadays it is, certainly, easier to set up a logging system for a MMS than twenty or even ten years ago.

### 3.2. Time Tagging/Synchronization

The great importance of an accurate time stamping of the data frames acquired by the sensors can be perceived in Table 1, which shows the travelled distance in small time intervals at typical velocities in land mobile mapping platforms. From the data in Table 1 it becomes evident that in order to ensure DGS accuracy of one decimeter we need to guarantee the millisecond level of accuracy in time synchronization. In most MMS works a brief explanation can be found about the way how the problem was handled [4,5,11,12].

Presently the Navstar GPS system is the best time reference that can be accessed, with an accuracy of the order of the nanosecond, with the advantage of being a simple and cheap method. Besides, this is the only feasible method that provides a direct, precise and safe time signal for MMS. There are two basic ways of synchronizing computer time with a GPS receiver. The first is based on the reception, from the GPS receiver, of NMEA (National Marine Electronics Association) messages that contain GPS Time. In this case the typical accuracy of a computer clock after being corrected will be between 1 and 10 milliseconds; the second uses the Pulse Per Second (PPS) from the GPS receiver that is a TTL (Transistor to Transistor Logic) signal type, synchronized with GPS s with great accuracy (around 40 nanoseconds). Since the PPS signal lacks absolute time information it is used together with NMEA time messages. In this case the synchronization accuracy of the computer clock, after being corrected, will be within 1 microsecond. The quality of computer time synchronization, using GPS time service is summarized in Table 2.

The 2 × 10^−2^ and 3 × 10^−4^ sums correspond to the mean drift of a software clock in one minute and in one second respectively. By observing Table 2 it can be concluded that only the synchronization with PPS and NMEA messages, with regular corrections every second, will lead to reliable computer time accuracy at the 1 millisecond level.

In general, the method relies on the transmission of GPS Pulse per Second (PPS) signal to the data logger where the data is being stored. A local text file will then register, at each received pulse, the drift of the computer clock during data acquisition. As the data frames are time tagged by the Operating System using computer time, that time is easily translated into GPS time by simply interpolating the drift file for the particular instants. A variation of this scheme consists in correcting the computer time at each PPS, directly accessing the BIOS, what avoids the need of generating the drift file and the latter interpolation.

### 3.3. System Calibration

The correct term to apply to the calibration of an MMS is system calibration because it implies some calibration procedures that are interrelated. DGS calibration, sensor calibration, relative orientation between sensors and relative orientation between DGS components and platform, are all aspects that have a role in the final quality of the achieved results.

Calibration of DGS components such as, Inertial Navigation Systems or digital compasses, will not be discussed in detail as those calibration parameters are usually embedded in the equipment and included in the solutions. As an example we can take the INS were gyro drifts and accelerometer biases are previously accessed by the manufacturer.

#### 3.3.1. Camera Interior Orientation

The characteristics and behavior of the image sensors are vital for the overall system performance. In one hand lens system must offer the possibility of iris and focal length fixing in order to keep the internal characteristics practically unchanged, at least during a surveying session, and, on the other hand, it must be possible to determine the internal characteristics by means of parameter estimation. The eight standard internal orientation parameters, presented by Brown [24], include lens focal distance, principal point location, radial distortion parameters (to model symmetric lens distortions related to the image principal point—approximately the image centre) and decentring distortion parameters to model asymmetric lens distortions.

The used methods are in general, classified as photogrammetric calibration and self-calibration. The former, which is associated with metric cameras, relies on images of a rigorously coordinated calibration object and allows, in general, better parameter estimation while being more robust. Self-calibration tries to obtain the calibration parameters with the smaller information possible, not using a calibration object, and is associated with computer vision and non metric digital video cameras. A general discussion of these methods can be found in [25].

**Figure 4.**
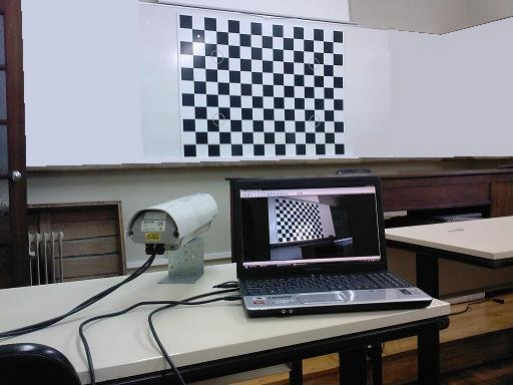
Image acquisition for camera interior orientation.

A good option in camera calibration is to use the approach described for example by Heikkila and Silven [26], or Zhang [27], which is a mixture of photogrammetric calibration and self-calibration through the use of a flat pattern as a calibration object. In doing so, the benefits of the photogrammetric method, jointly with a greater level of simplicity and speed of operation can be explored. For each camera several images must be obtained, from different angles, of an object with well defined points that must lie in a plane pattern like the one shown in Figure 4.

#### 3.3.2. Relative Orientation between Remote Sensors

**Figure 5.**
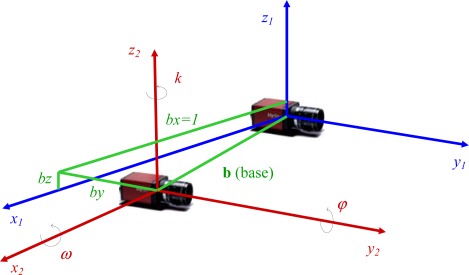
Relative orientation between two cameras.

The relative positions and rotations between MMS components have to be known with sufficient accuracy. For each component pair six relative orientation parameters are defined: three translations and three rotations. Figure 5 shows an example of relative orientation parameters between two video cameras.

The translations are the three base components (*b_x_*,*b_y_* and *b_z_*) and the rotations are the anti-clockwise rotation angles of the second camera relatively to the first camera 3D referential. Namely the *ω*, *φ* and *κ* angles are, respectively, the X, Y and Z axis rotations. The way how these parameters can be obtained, in the case of video cameras, relies in photogrammetry, were two images of the same scene obtained by the cameras can be interpreted in order to extract those parameters.

#### 3.3.3. Relative Orientation between Sensors and Direct Georeferencing System

**Figure 6.**
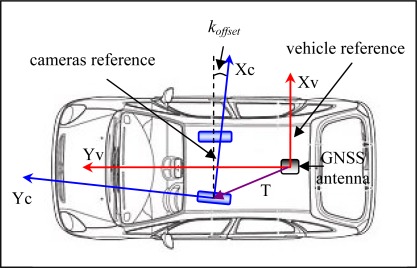
Cameras and vehicle reference frame.

Besides the relative orientation parameters between remote sensors it is also necessary to obtain the relative orientation between one of them and the DGS referential. It can be assumed that the DGS reference system axes match those of the moving platform and its center coincides with the INS origin or with the phase center of a GNSS receiver. In this case the parameters are called angular and linear offsets. In Figure 6 an example is presented, showing only the XY plane. In this case the DGS centre is the phase centre of a GNSS antenna and the v subscripts stands for “vehicle”, while the c subscripts for “camera”. The linear offsets are the three components of the translational vector T. As the figure is depicted in a horizontal plane only the rotation of the Z camera axis relatively to the DGS can be observed, named the *κ_offset_*.

One way to obtain the offset parameters relies on a photogrammetric process called space resection [28], that needs external control points, jointly with good DGS estimates. Space resection allows for the determination of the positions and attitudes of the cameras in different instants, with image coordinates of control points as input. The positions and attitudes of the cameras are then compared with the positions and attitudes of the DGS for the same instants.

It must be stressed that the parameters that need space resection to be obtained are angular attitudes, which have a greater role in error propagation. A simpler way of obtaining the angular offsets between a camera and the DGS, which relies on the Relative Orientation process itself, and some initial assumptions, is presented in Section 4.

Linear offsets can be obtained by means of a dedicated topographic survey or measuring with a metric tape (depending on required accuracy). The remaining associated errors, usually very small, are constant in the process of MMS acquisition of coordinates.

### 3.4. Acquiring Georeferenced Information from the Remote Sensors

The final goal of a MMS is to obtain georeferenced information of objects captured by the sensors, despite the fact that qualitative information can, also, be acquired. In a first stage the data collected during a field survey has to be stamped with its position and attitude. This is done by means of software, linking all the data using a hinge that is GPS or local time. Usually a high order interpolation method is used because DGS and remote sensors work, normally, at different rates. The Relative Orientation parameters and offsets, already issued in previous sections, are also used in this step in order to translate coordinates between components. The second stage consists in obtain georeferenced positions of objects observed in the images, using positions and attitudes tagged to the images in the previous stage.

**Figure 7.**
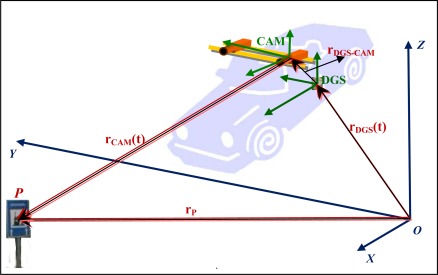
Scheme of point georeferencing in a MMS.

In Figure 7 the complete scheme that rules the georeferencing of points captured by the remote sensors is presented. In this case the sensors used were two digital cameras and the DGS is an integrated GNSS/IMU system.

The components presented in the scheme of Figure 7 are the following:

- The absolute coordinate system, (*X*, *Y*, *Z*) where the coordinates of an object point, *P*, are intended to be obtained.
- The coordinate system associated with the DGS-DGS reference system.
- The coordinate system associated with the cameras-CAM reference system.
- The absolute position vector of the object point *P*, **r_p_**
- The absolute position of DGS centre in *t* instant, **r**_DGS_(t).
- The position and attitude of cameras referential related to DGS reference system. These are called the linear and angular offsets. In the figure the linear offset is **r**_DGS-CAM_ and the angular offsets will be represented by the matrix **M**_DGS-CAM_.
- Finally the position of point *P* in cameras reference system, **r**_CAM_(t).

Other needed elements, not showed in the figure are the attitude angles of DGS in *t* instant, that will be represented as the rotation matrix **M**_DGS_*(t)*.

Considering a scale factor between DGS and cameras reference systems of *s*, the absolute position of object point *P* will be computed as:

(1) $${\vec{r}}_{P}=r_{\mathrm{DGS}}(t)+M_{\mathrm{DGS}}(t)\times r_{\mathrm{DGS}-\mathrm{Cam}}+s(M_{\mathrm{DGS}}(t)\times M_{\mathrm{DGS}-\mathrm{Cam}})\times r_{\mathrm{Cam}}$$

The above formula states that the remote sensors of a MMS allow, in fact, to extend the acquisition of georeferenced information to objects surrounding the vehicle path.

## 4. The Low Cost System Developed at University of Porto

In this section the system developed by the authors is presented. The main purpose was to develop a system that could be adapted to different environments using low cost equipment and keep in an acceptable level the overall complexity in surveying procedures and system calibration. The only fixed module is the image acquisition system which uses a current consumer laptop, two small format digital colour video cameras and a modified GPS receiver in order to accurately time tag the images. We start by presenting these components in the next sections.

User friendly software was also purposely developed, using Matlab programming language, to automatically perform some usual tasks such as finding relative orientation parameters between cameras, getting coordinates or measure captured object dimensions.

### 4.1. The GPS-CAMSYNC Unit

A fixed component in the developed system is based on a U-blox receiver board, which is well adapted for urban environments. This board allows for continuous, smoothed, solutions even in bad GNSS visibility, as it contains a low cost gyroscope, connection to the car odometer and for forward/reverse indication. A navigation integrated solution is calculated in real time using an internal enhanced Kalman filter. If no other system is available this unit can be used as DGS.

**Figure 8.**
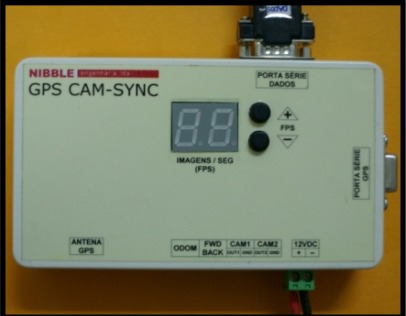
GPS CAM-SYNC box.

The described GPS receiver board was enclosed in a box, named GPS CAM-SYNC (Figure 8), and a frequency multiplier was added to the pulse per second, allowing frequencies between 2 and 30 PPS.

One of the main tasks of the GPS-CAMSYNC box is to generate a GPS synchronized time frequency, changeable with two buttons in the outside of the box (see Figure 8), to trigger the cameras.

The navigation data, stored in a flash memory once per second, is composed by WGS84 latitude and longitude, height, car velocity and time in UTC (Universal Time Coordinated) format. The instantaneous vehicle heading is derived from the GPS trajectory as it is allowed by its smoothness. Horizontal accuracy, as indicated by the manufacturer, is around two meters which is sufficient for many kinds of road infrastructure inventory.

### 4.2. Image Acquisition Module and Synchronization with GPS Time

**Figure 9.**
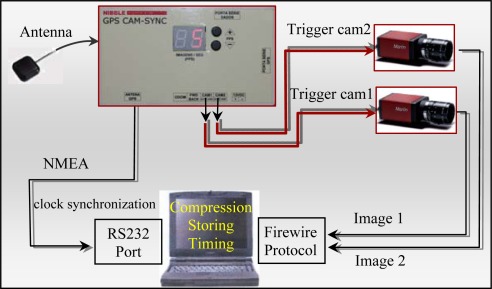
Image acquisition architecture.

Cameras are connected to the laptop through a Firewire card port and the images are stored in JPEG format (a complete scheme is presented in Figure 9). It is necessary to get a precise time for each acquired image to precisely discriminate their position and attitude in the absolute reference system. The external trigger possibility offered by the cameras is used in conjunction with the frequency generated by the GPS CAM-SYNC which is, as described before, synchronized with the GPS PPS. In this way, the perfect simultaneity of the frames acquired by both cameras and its accurate synchronization with GPS time is guaranteed. Time precision of the used receiver board PPS is about 50 nanoseconds (as indicated by the manufacturer) and the same is expected for the trigger requests.

To correctly time tagging the images, the system time of the logging computer must be also synchronized with GPS time, although not so accurately as is intended for the images itself. The chosen procedure was to use the same receiver that is triggering the cameras to synchronize the laptop with real time NMEA messages, through a RS232 connection port, using current commercial software. This kind of laptop time synchronization typically leads to a 0.01 seconds of clock accuracy in the laptop, as showed in Table 2. Furthermore, at maximum frame rates of 10 Hz, real time compression and storing by an up to date laptop is feasible using a Firewire protocol card port.

To improve the correct time tagging of the acquired frames it was decided that the frequency generated by the GPS CAM-SYNC will miss the pulse corresponding to the integer second. In this way, the longer time intervals between acquired images allow to identify where the integer seconds are.

It is important to notice that image time tagging is not performed during acquisition. The data logger’s role in this system is only to allow for the discrimination of the integer second once the fractional part is already known. Time tagging is executed in post-processing using a software module specifically developed for this task.

### 4.3. Calibration Procedures

As stated previously, calibration of a MMS comprises sensor calibration and determination of relative positions and orientations between components. As these parameters remain unchanged at least during a survey event, they can be settled accurately prior to the survey.

Camera interior orientation is performed independently for each camera. The method used was described in Section 3.3.1, but can be reviewed in more detail in reference [29]. It relies on images of the flat pattern presented in Figure 4. Initial approximations for the exterior parameters, three rotations and three translations for each image in the object space, are not needed once they are calculated on a stage inside the method. Final orientation parameters are obtained from an iterative process using a bundle adjustment.

Relative orientation between cameras relies also on a photogrammetric bundle adjustment whose input are the image coordinates of conjugate points in a photogrammetric pair. As an innovative aspect, the authors developed a modification in the standard collinearity equations, by using a control distance, measured in the object space that is introduced as a constraint in the set of equations as a way to overcome relative orientation angular instability due to small distance between cameras. This means that the distance between one or two of the observed points and the origin of cameras referential (see Figure 5) is measured with a laser distance meter and the calculated relative coordinates for these points must verify the measured distances. As a result all the relative orientation parameters has to adapt to this constraint, including *k* angle (Figure 5), for which instability was observed, being this angle mandatory in the measured distances to object points. This approach revealed excellent results because in all the experiments it could be observed that the accuracy of the obtained distances was significantly improved, in general by a factor of ten.

Other innovative aspect in the presented work is the linkage of the determination of angular offset parameters between DGS and cameras reference systems to the process of relative orientation between cameras, described in the previous paragraph. Based on Figure 6 and assuming perpendicularity between vehicle axis *Yv* and the cameras base vector, the *φ* and *κ* *offset* parameters (rotations of *Y* and *Z* camera axis relatively to the vehicle reference system) are obtained with the following expressions:

(2) $$\varphi_{\mathrm{offset}}=\text{arcsin}\frac{\mathrm{Tz}}{B},\;\varphi_{\mathrm{offset}}\;\in \;\left[-\frac{\pi }{2},\;\frac{\pi }{2}\right]$$

(3) $$k_{\mathrm{offset}}=\text{arcsin}\frac{\mathrm{Ty}}{-\text{cos}\;\varphi .B},\;\kappa_{\mathrm{offset}}\;\in \;\left[-\frac{\pi }{2},\;\frac{\pi }{2}\right]$$

where *Ty* and *Tz* are the linear relative orientation parameters between cameras, respectively in the *Y* and *Z* axis, and *B* is the length of the base vector between cameras. This process allows one to obtain good approximations for κ*_offset_* and φ*_offset_*, however it did not prove to be robust enough and the parameters have to be refined using control points in the field. However this avoids more complicated schemes, as the need to perform space resection with field control.

## 5. Tests and Results

Several tests were performed in order to perceive the main role of each piece of the system in the quality of the acquired georeferenced information and, also, assess the overall accuracy that the system could achieve.

### 5.1. Test of System Error Propagation in Static Mode

**Figure 10.**
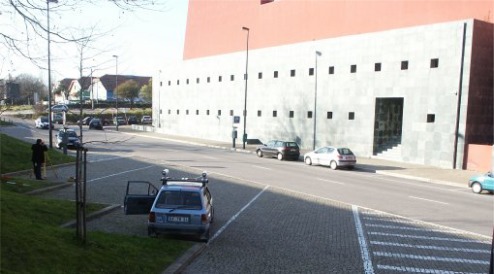
Total station survey for tests in relative coordinates and distances.

A test to assess the extent of error propagation that is introduced by the videogrametric system was carried out. The system was mounted without a DGS and the remote sensors used were two video cameras, with characteristics adapted to the implemented system, namely: digital acquisition through a color CCD (charged couple device) of ½ inch, resolution of 640 × 480 pixels, progressive scan, transfer and control through Firewire protocol, external asynchronous trigger shutter. The lens systems that equip the cameras are C mount, high resolution, with 12 mm focal length (which can be considered a standard lens). To implement the test, the system was mounted on a stopped vehicle in front of a building façade (Figure 10). Points on this façade were surveyed with a total station, as well as the coordinates of perspective centers of cameras (at 1 cm precision) and the correspondent attitude angles. The process was done in three locations of the vehicle from which 32 points of known coordinates in the building and its surroundings were measured with the system in all of its positions. The coordinates of perspective centers of cameras (at 1 cm precision) and the correspondent attitudes were also determined using the process described in Section 4.3.

The statistical analysis (mean and RMS error) of the obtained coordinates are presented in Table 3.

A division was made between distances between 10 and 21 m and between 21 and 31 m. At this point it was observed that the errors introduced by the calibration processes and by the determination of the conjugate image points are very well self contained, leading to errors in measured coordinates, in general smaller than 15 cm at distances of about 21 m or less. For distances between 21 and 31 meters errors increase but are in general less than 25 cm. However a systematic error could be observed, that increases with distance, that authors attribute to a lack of strength in the method used to obtain the angular offsets. Could this systematic error be eliminated and the results certainly improve.

### 5.2. Test in Absolute Coordinates Survey (a)

**Figure 11.**
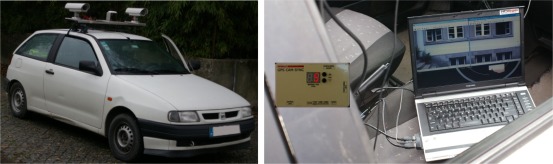
System components during survey.

In the previous test errors introduced by the DGS were not present. This second test will account for those errors. The system was setup in the simpler way that it can be. The used DGS was the GPS-CAMSYNC box and the cameras used were the same as in the test described in the previous section. System components, when in surveying, are represented in Figure 11.

Twenty traffic signs distributed in urban and near urban areas, were surveyed with GNSS differential static methods. The accuracy of those measurements is expected at centimeter level. With the described MMS mounted on a vehicle this traffic signs were surveyed when at distances between 10 and 45 meters. Differences with the GNSS coordinates were calculated and are presented in Table 4, together with the mean and the RMSE.

**Figure 12.**
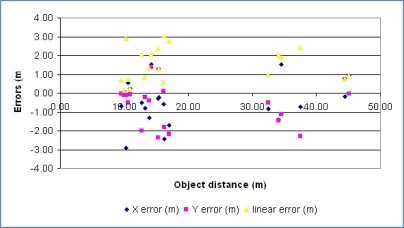
Errors in measured absolute coordinates with distances to objects in meters.

The positioning method of the DGS uses only pseudo-ranges and EGNOS corrections. The expected accuracy, *i.e.*, RMS errors near or under 2 meters, was exceeded, what can be attributed to severe GNSS observation conditions. The errors introduced by the videogrammetry system, as shown before, are of smaller magnitude. The graph in Figure 12 shows the X, Y and linear errors against the distance to the coordinated point. It can be observed that there is not an evident connection between errors and distances to the coordinated objects.

It is clear to the authors that the errors introduced by the direct geo-referencing system, actually the data from the GPS CAM-SYNC box, are contributing with the larger portion of the final errors in coordinates and masking the smaller errors introduced by the cameras system, which increases with the distance.

The mean of the Y error is high, indicating a systematic error that could not be identified once the survey trajectories, when measuring object coordinates to this experiment, occurred practically in all directions, so it cannot be a shift in the measured distance or in *κ* attitude angle.

### 5.3. Test in Absolute Coordinates Survey (b)

In another test a better DGS was used, that consists in a Crossbow IMU of 3 axis and a Novatel dual-frequency GNSS phase receiver, which allows one to obtain DGS loosely coupled solutions by means of a Kalman filter. The cameras used were of the same type of those used in the previous two tests, however in this case the color CCD are of ¼ inch and the lens systems that equip the cameras has a focal length that can vary between 1.8 and 3.6 mm. This means that these cameras are very wide angle, allowing observing objects at much closer distances. Twenty two control points, consisting of traffic signs and points on the road were surveyed in a wide open area, at ranges between 4 and 10 m. A differential GNSS phase solution was obtained from the GNSS receiver for most of the surveyed points. The results of the differences from the solution obtained with the MMS are presented in Table 5.

It must be stressed that the offset angles obtained with the method described in Section 4.3 were used as initial approximations and the final offset angles were obtained after correction of the initial approximations using control points. Other important aspect is that the used cameras allowed surveying the objects at much closer distances, in so minimizing the propagation of errors in the offset angles. This test showed that the system can work at different accuracy levels depending on the type of equipment that is used at each particular application.

## 6. Conclusions

In order to implement a land Mobile Mapping System there are a set of inter-dependant aspects that must be considered. Our main goal was to keep in simple level technical and equipment demands, while trying to achieve good quality standards in the final results.

In a MMS a great role is played by the sensors that integrate different system components. In the present case a low cost DGS composed by a single frequency GNSS receiver, a one axis gyroscope and connection to car odometer is permanently present in the system, allowing for a good quality, smoothed, georeferenced trajectory. However other DGS can be used if are available and it is justifiable by a particular application that needs more accuracy in the acquired coordinates.

As remote sensors, digital low cost cameras were used with characteristics adapted to the system, namely small format images, lens with the possibility of rigidly fix its internal parameters and external trigger device.

To deal with time synchronization of acquired data cameras with trigger were chosen, using a pulse linked to the PPS of the GNSS receiver on the DGS, overcoming in this way two important concerns in mobile mapping: first the simultaneity of acquired frames by both cameras, and secondly, the correct discrimination of the GPS acquisition time with an accuracy close to the milliseconds.

For processing and storing acquired data in real time an up to date laptop was used. Its architecture, added with a Firewire protocol for data transfer, showed to be well adapted to the high amount of transferred, processed and stored data during a MMS survey.

The methods presented for camera calibration and relative orientation between sensors components, maintain good standards in the accuracy of the parameters obtained while avoiding complicated schemes, mainly in offset determination between DGS and cameras reference systems, once that process was linked to relative orientation between the cameras.

Tests have shown that the videogrammetry system, in which the coordinates of points are obtained in cameras reference frame, contributes to the positional accuracy with an error that, increasing with distance, remains below three decimeters to distances up to 30 m. However this test shows also that the accuracy of the videogrammetric system is influenced in a systematic way by errors in offset angles. The improvement of the methodology used to obtain the offset angles certainly lead to a less error propagation from the videogrammetric system. Direct Georeferencing System is one of the main bottlenecks in the overall accuracy, especially in urban areas were GNSS observation conditions are poor.

More sophisticated existing systems, referred in the introduction, while quite efficient have the disadvantage of being very expensive. This modular system has advantages in terms of cost and usability, while still providing an accuracy which is enough for most of road infrastructure survey that is compatible with medium and small scale representation.

## Figures and Tables

**Table 1.** Travelled distance related to velocity.

**Time**	**40 km/h**	**60 km/h**	**100 km/h**
**1 second**	11 meters	17 meters	28 meters
**1 centesima**	0.11 meters	0.16 meters	0.28 meters
**1 milisecond**	0.01 meters	0.02 meters	0.11003 meters
**1 microsecond**	1 × 10^−5^ meters	2 × 10^−5^ meters	3 × 10^−5^meters

**Table 2.** Computer time accuracy.

**Type of Time keeping**	**Computer Time accuracy**
Autonomous mode	Typically 2 to 5 seconds per hour after being turned on.
With regular corrections (1 min) by GPS (just NMEA)	10^−3^ to 10^−2^ + 2 × 10^−2^ s
With regular corrections (1 min) by GPS (NMEA + PPS)	10^−6^ + 2 × 10^−2^ s
With regular corrections (1 s) by GPS (just NMEA)	10^−3^ to 10^−2^ + 3 × 10^−4^ s
With regular corrections (1 s) by GPS (NMEA + PPS)	10^−2^ + 3 × 10^−4^ s

**Table 3.** Mean and RMS error in object coordinates of the 32 points measured with total station.

	**10 m < Distances < 21 m**		**21 m < Distances < 31 m**	

	**X error**	**Y error**	**X error**	**Y error**
Mean	−0.02 m	−0.02 m	0.13 m	−0.12 m
RMS	0.12 m	0.07 m	0.21 m	0.12 m

**Table 4.** Errors obtained in absolute coordinates of 20 traffic signs.

	**X error (m)**	**Y error (m)**	**Linear error (m)**
Mean	−0.18	−1.48	2.17
RMSE	1.37	1.96	2.39

**Table 5.** Errors obtained in absolute coordinates of 22 traffic signs.

	**X error (m)**	**Y error (m)**	**Linear error (m)**
Mean	0.02	−0.01	0.22
RMSE	0.27	0.16	0.31
